# Additive Interaction between Heterogeneous Environmental Quality Domains (Air, Water, Land, Sociodemographic, and Built Environment) on Preterm Birth

**DOI:** 10.3389/fpubh.2016.00232

**Published:** 2016-10-24

**Authors:** Shannon C. Grabich, Kristen M. Rappazzo, Christine L. Gray, Jyotsna S. Jagai, Yun Jian, Lynne C. Messer, Danelle T. Lobdell

**Affiliations:** ^1^National Health and Environmental Effects Research Laboratory, U.S. Environmental Protection Agency, Research Triangle Park, Chapel Hill, NC, USA; ^2^National Health and Environmental Effects Research Laboratory, Oak Ridge Institute for Science and Education, U.S. Environmental Protection Agency, Chapel Hill, NC, USA; ^3^Gillings School of Global Public Health, University of North Carolina, Chapel Hill, NC, USA; ^4^Division of Environmental and Occupational Health Sciences, School of Public Health, University of Illinois Chicago, Chicago, IL, USA; ^5^School of Community Health, College of Urban and Public Affairs, Portland State University, Portland, OR, USA

**Keywords:** environmental quality, interaction, air, water, sociodemographic, built environment, land, preterm birth

## Abstract

**Background:**

Environmental exposures often occur in tandem; however, epidemiological research often focuses on singular exposures. Statistical interactions among broad, well-characterized environmental domains have not yet been evaluated in association with health. We address this gap by conducting a county-level cross-sectional analysis of interactions between Environmental Quality Index (EQI) domain indices on preterm birth in the Unites States from 2000 to 2005.

**Methods:**

The EQI, a county-level index constructed for the 2000–2005 time period, was constructed from five domain-specific indices (air, water, land, built, and sociodemographic) using principal component analyses. County-level preterm birth rates (*n* = 3141) were estimated using live births from the National Center for Health Statistics. Linear regression was used to estimate prevalence differences (PDs) and 95% confidence intervals (CIs) comparing worse environmental quality to the better quality for each model for (a) each individual domain main effect, (b) the interaction contrast, and (c) the two main effects plus interaction effect (i.e., the “net effect”) to show departure from additivity for the all U.S. counties. Analyses were also performed for subgroupings by four urban/rural strata.

**Results:**

We found the suggestion of antagonistic interactions but no synergism, along with several purely additive (i.e., no interaction) associations. In the non-stratified model, we observed antagonistic interactions, between the sociodemographic/air domains [net effect (i.e., the association, including main effects and interaction effects) PD: −0.004 (95% CI: −0.007, 0.000), interaction contrast: −0.013 (95% CI: −0.020, −0.007)] and built/air domains [net effect PD: 0.008 (95% CI 0.004, 0.011), interaction contrast: −0.008 (95% CI: −0.015, −0.002)]. Most interactions were between the air domain and other respective domains. Interactions differed by urbanicity, with more interactions observed in non-metropolitan regions.

**Conclusion:**

Observed antagonistic associations may indicate that those living in areas with multiple detrimental domains may have other interfering factors reducing the burden of environmental exposure. This study is the first to explore interactions across different environmental domains and demonstrates the utility of the EQI to examine the relationship between environmental domain interactions and human health. While we did observe some departures from additivity, many observed effects were additive. This study demonstrated that interactions between environmental domains should be considered in future analyses.

## Introduction

Environmental exposures such as pollutants, social factors, and built environment likely have a collective influence on health; however, epidemiological research often focuses on singular exposures. This may be in part due to the complexity of measuring multiple environmental factors in tandem (1, 2). Some research, including air pollution studies, has used indices or decomposition methods such as principal component analysis (PCA) to assess the simultaneous impact of different pollutants (3). These methods are also seen in built and social epidemiological research in the assessment of neighborhood effects or total social constructs (4–6). While indices are becoming more widely used across epidemiology, very few studies have combined variables across separate environmental domains (e.g., air, water, and built environment). The assessment of cumulative environmental exposure is currently being targeted as a need in epidemiological research (7).

Interaction is the concept that the effect of one exposure may depend partly on the presence, absence, or level of another exposure (8). One might expect that two detrimental exposures occurring simultaneously would potentially lead to a more detrimental effect than a single exposure on a given outcome; however, this is not always the case. Interaction can be defined by a departure from multiplicativity (on the log or logit scales such as those used in risk or odds ratio estimation) or from additivity (on the linear scale used to estimate difference measures) (9). Interaction on the additive scale is often thought of as more relevant to public health because the additive scale reflects absolute numbers of persons rather than relative risks or odds (as in the multiplicative scale), which may only translate to change in few individuals (10). One exposure may enhance the effects of another, creating a synergistic interaction, or may diminish the effects of another, creating an antagonistic interaction. In the case of two detrimental environmental exposures, a synergistic interaction would indicate a much worse health effect overall, while antagonistic interaction would result in estimates that are closer to or even across the null value, appearing as beneficial effects. For two exposures in which both have an independently beneficial effect, antagonism would result in a less beneficial association than expected (i.e., less than a strictly additive effect) and synergism would create an even more beneficial association than expected. If two exposures have effects in opposite directions (i.e., one is beneficial and the other is detrimental), then antagonistic effects would be less than expected, which could be closer to or away from the null, depending on which exposure is considered the “main effect.” Interaction is a manifestation of the complex processes giving rise to illness and health. Exploration of interactions may lead to better understanding of these processes.

Though it is important to study the potential effects of interaction, such studies are often challenging to undertake. Analyses of interaction inherently have less power than those of main effects. Interactions do not always follow a simple form, i.e., effects of exposure may differ in a non-linear fashion across levels of another factor or may differ only at certain levels of the factor, making interpretation difficult. While statistical interaction is what one can estimate, connecting statistical to biological or public health interaction requires substantive knowledge of how biological systems can be influenced by exposures in the presence of one another. While recently many epidemiological studies have explored gene–environment interactions, none has assessed interactions across environmental domains (11). Currently, only one metric, the Environmental Quality Index (EQI), has been developed to assess both domain-specific and cumulative environmental exposure. The EQI is a publically available county-level measure of cumulative environmental exposures for the U.S. for the period 2000–2005 (2, 12). The EQI includes variables representing five environmental domains: air, water, land, built, and sociodemographic. The index provides a cumulative total measure of the ambient environment and domain-specific indices.

The EQI has been used as an exposure to assess association between environmental quality and several health outcomes. In a study of preterm birth (PTB), the authors observed positive associations between the air domain and PTB across all rural/urban strata and for the sociodemographic domain in the most urban stratum, and null or negative associations between the other domains and PTB, and the overall EQI and PTB (13). A study of mortality generally found worse environmental quality to be positively associated with mortality (14). One study applied the EQI to control for environmental confounding in the association between hurricanes and reproductive health outcomes (15). While the EQI allows researchers to consider multiple environmental constructs simultaneously, no published studies have examined potential interactions between environmental domains in the assessment of human health.

We address this current gap in the literature by conducting a county-level cross-sectional analysis of interactions between EQI domain indices on PTB in the Unites States from 2000 to 2005. We used PTB as the motivating example in this analysis as it is a marker of fetal underdevelopment and a risk factor for further poor health outcomes (16–19), can be used as an indicator of national health (20, 21), and to further develop the previously published analysis (13). In addition, we stratified by urban/rural status as it has been shown that the association with PTB and interactions can vary greatly by urbanicity (22).

## Materials and Methods

### Study Population

The study population for this analysis has been previously described in Rappazzo et al. (13). In brief, the study population included live births from the National Center for Health Statistics (NCHS) for the entire United States for the years 2000–2005 for all 3141 counties. The study population was restricted to singleton, non-anomalous births, with county identifiers, recorded gestational age, and residence within the same state as birth occurrence (*n* = 22,705,068). County-level PTB prevalence was estimated as PTBs/total births for all 3141 U.S. counties. PTB is defined as birth occurring between 20 and 36 weeks completed gestation (inclusive). Ten counties were excluded because less than 10 total births or no PTBs occurred over the study period, leaving a final population of 3131 counties. The study protocol was reviewed by the EPA Human Research Protocol Office and deemed non-human subject research as per EPA Regulation 40 CFR 26 (Protection of Human Subjects) Section 26.102 (f).

### Environmental Quality Index

Domain-specific EQIs were used to represent environmental exposure at the county-level for the entire U.S. over the 2000–2005 time period. The EQI includes variables representing five environmental domains: air, water, land, built, and sociodemographic (2). The domain-specific indices include both beneficial and detrimental environmental factors. The air domain includes 87 variables representing criteria and hazardous air pollutants. The water domain includes 80 variables representing overall water quality, general water contamination, recreational water quality, drinking water quality, atmospheric deposition, drought, and chemical contamination. The land domain includes 26 variables representing agriculture, pesticides, contaminants, facilities, and radon. The built domain includes 14 variables representing roads, highway/road safety, public transit behavior, business environment, and subsidized housing environment. The sociodemographic environment includes 12 variables representing socioeconomics and crime.

Briefly, the EQI was constructed using a separate PCA for each of the five environmental domains, and the primary component was retained to represent that domain’s index. The primary component for each domain was then used in a subsequent PCA to create the overall EQI for total environmental quality. The EQI and domain-specific index construction was also stratified by condensed rural/urban continuum codes from the United States Department of Agriculture Economic Research Service (23) as has been described in prior research (24–26): metropolitan urbanized, non-metropolitan urbanized, less urbanized, and thinly populated. Full methods of the EQI’s construction are described in Ref. (2), while data description can be found in Ref. (12).

### Data Analyses

County prevalence of PTB was defined as the proportion of PTBs among all live births in each county for 2000–2005. The exposure variables for the analyses were urban/rural-stratified domain-specific indices. Index values were linked to PTB prevalences by maternal county of residence. Domain indices were categorized by tertiles where lower values indicated better environmental quality, midrange values indicated average, and higher values indicated worse environmental quality. Linear regression was used to estimate prevalence differences (PDs) and 95% confidence intervals (CIs) for average (second tertile) and worse (third tertile) quality tertile compared to better tertile (first tertile) of environmental quality as the referent group.

**Figure 1:**
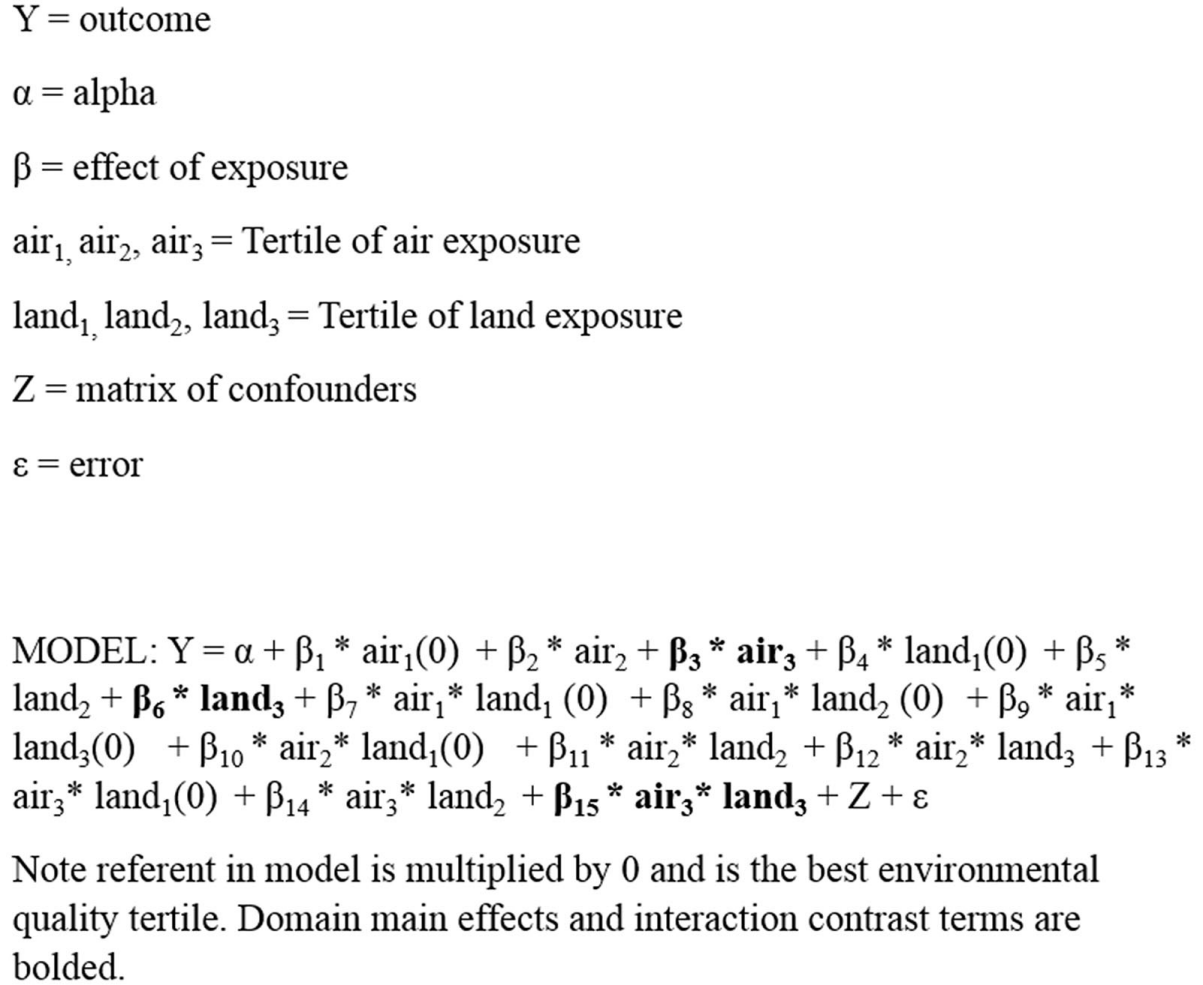
**Statistical model form: county-level linear model with domain interaction (example air by land)**.

Analyses compared worse environments to better environments. Therefore, positive PDs indicate an increase in PTBs with worse environmental quality, while negative PDs indicate a decrease in PTBs with worse quality, with a null value at 0. Comparisons were conducted in this manner to align with the previously published paper (13). Domain main effects and interaction effects were included for each tertile combination using two independent domains at a time. An example statistical model presented in Figure 1, using air and land domains as an example, displays how the air and land domains would have three levels each, with the lowest level acting as the referent [shown by (0)], and terms for interactions between domains at each level. As we estimated PDs (as opposed to an odds or risk), the interaction is assessed on the additive scale. A conservative interaction *p*-value of <0.05 was used to assess departure from additivity.

Models were also simultaneously adjusted for the EQI domains which were not included in the interaction (e.g., if the interaction was between water and air, we adjusted for land, sociodemographic, and built environment). Models included a percent minority covariate to account for differences between race and preterm delivery, as well as county-level confounding due to the non-random distribution of environmental disamenities (27). This schema included 10 models for each urban/rural category to include all interactions between the 5 respective domains.

Results are presented as PD and 95% CI comparing worse environmental quality to the better quality for each model for (a) each individual domain main effect, (b) the interaction contrast, and (c) the two main effects plus interaction effect (i.e., the “net effect”) to show departure from additive interaction. This net effect is the cumulative association between the two interacting domains on PTB prevalence.

### Ethics Statement

This research was approved by the U.S. EPA’s Human Subjects Review Office under Exempt Category 45 CFR 46.101(b).

## Results

**Figure 2:**
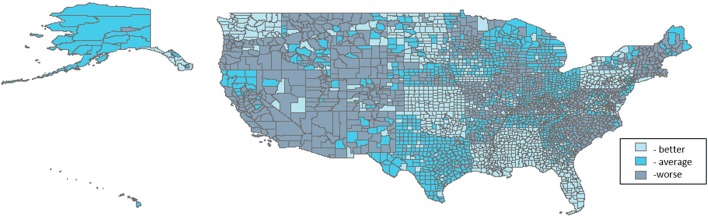
**Tertile distribution of the water domain (2000–2005) across U.S. counties**.

**Figure 3:**
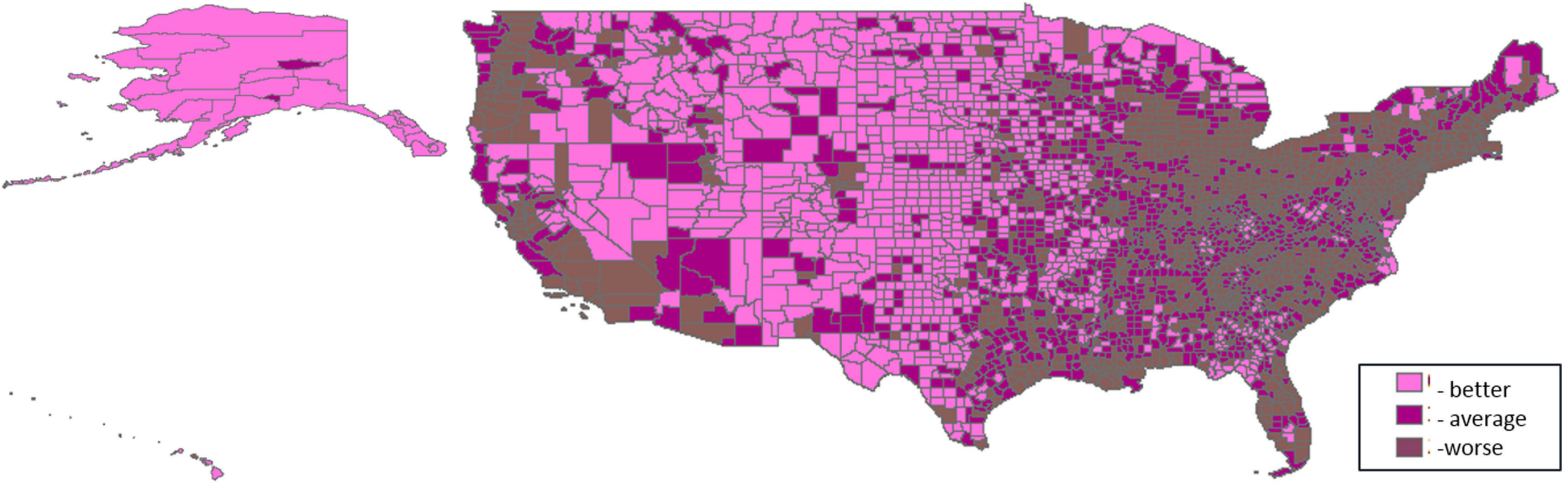
**Tertile distribution of the air domain (2000–2005) across U.S. counties**.

**Figure 4:**
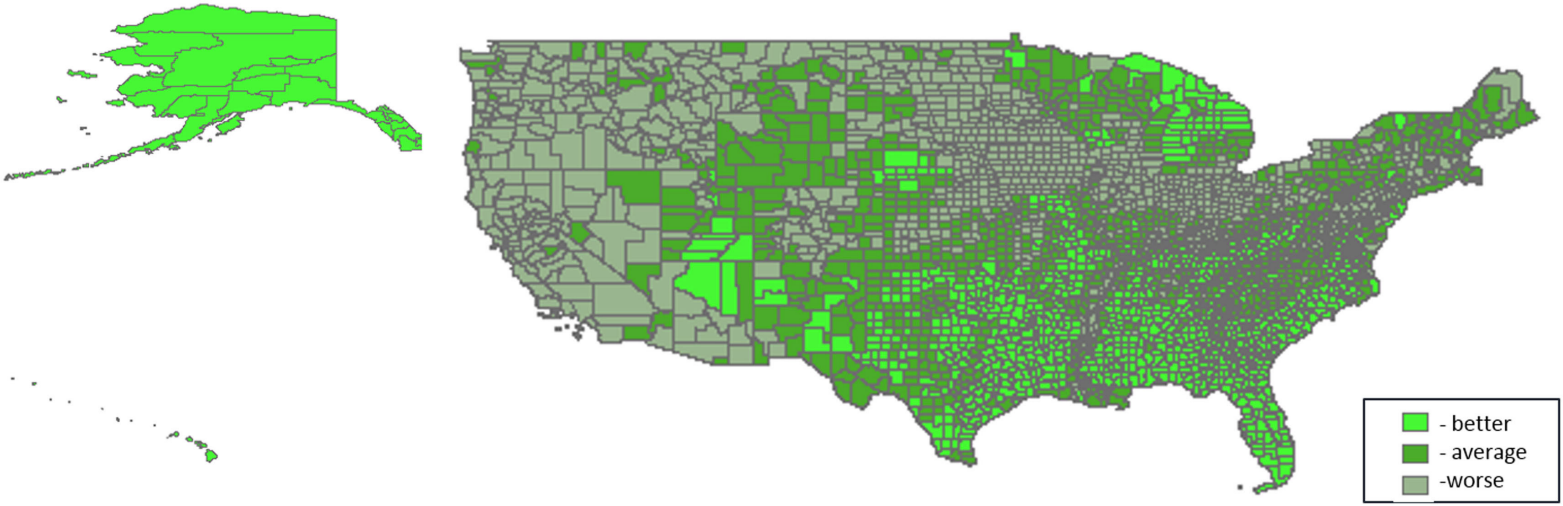
**Tertile distribution of the land domain (2000–2005) across U.S. counties**.

**Figure 5:**
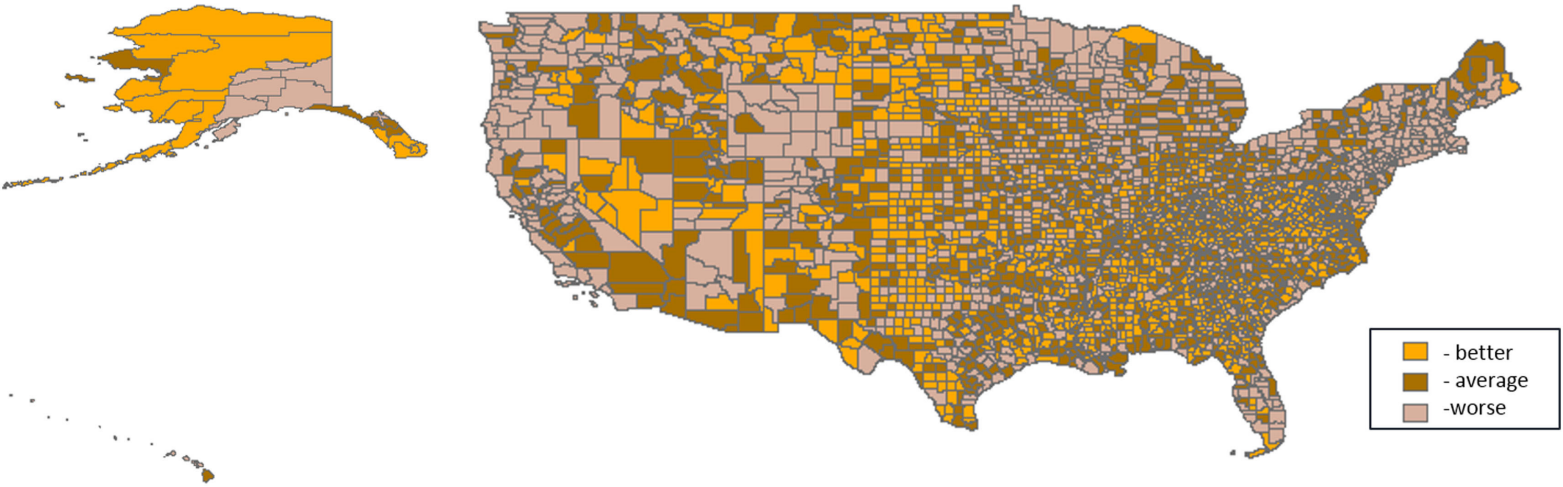
**Tertile distribution of the built domain (2000–2005) across U.S. counties**.

**Figure 6:**
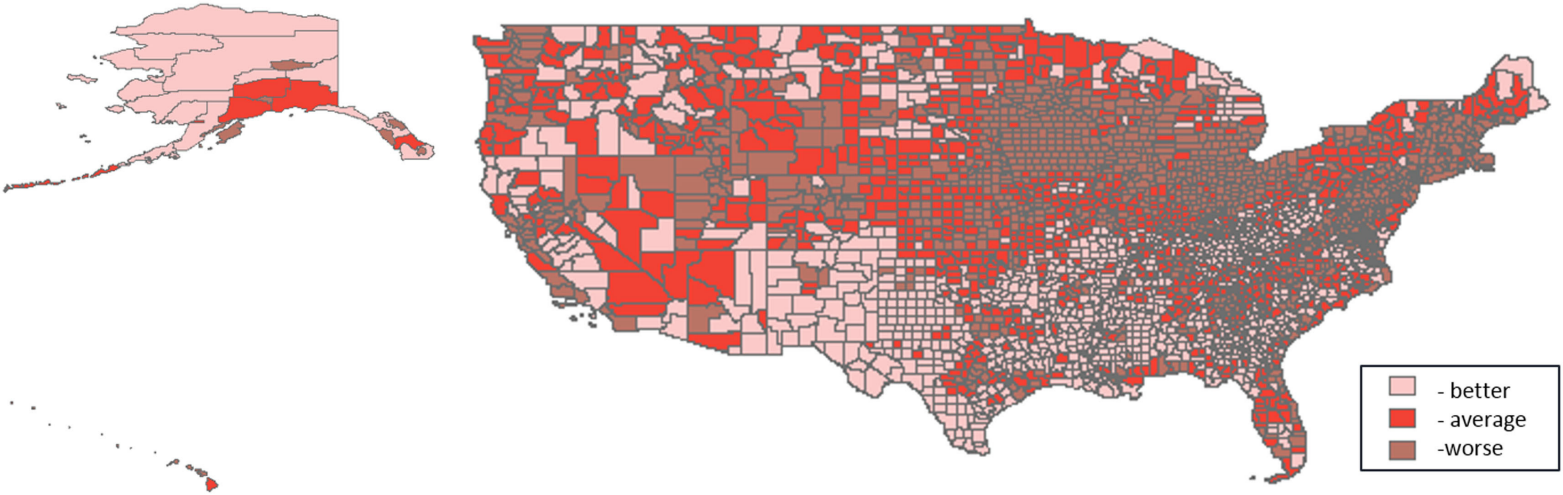
**Tertile distribution of the sociodemographic domain (2000–2005) across U.S. counties**.

Three thousand one hundred and thirty-one U.S. counties were included in the analyses. Distributions of PTB are shown in Table 1. For all counties, prevalence of PTB ranged from 1.85 to 60.56%, and percent minority population ranged from 0.69 to 97.39%. Patterns of the domain-specific tertiles varied spatially (Figures 2–6). Visually, particular domains exhibit similarity in spatial patterns of worse, average, and better environmental quality; for example, the water and land domains have distributions that resemble one another, while the air built and sociodemographic domains appear to be similar to each other. Tables 2–6 describe the main effects for each domain (PD and 95% CI), the interaction contrast term (e.g., air × land only), and the net effect (e.g., PD of air + land + air × land) from the overall non-stratified (entire U.S.) and the four urban/rural-stratified models.

**Table 1:** **Distributions of preterm birth (PTB) prevalence for 3141 U.S. counties, by urban/rural strata**.

Statistic	All counties	Metropolitan urbanized counties	Non-metro urbanized counties	Less urbanized counties	Thinly populated counties
Minimum	0.0185	0.0328	0.0533	0.0331	0.0185
25th percentile	0.0842	0.0868	0.0865	0.0859	0.0741
Median	0.1012	0.1000	0.1033	0.1047	0.0984
75th percentile	0.1201	0.1145	0.1183	0.1246	0.1220
Maximum	0.6056	0.6056	0.1931	0.2967	0.2500
Mean	0.1041	0.1033	0.1048	0.1072	0.0998
SD	0.0301	0.0273	0.0252	0.0306	0.0370

**Table 2:** **Prevalence differences^a^ (95% confidence interval) for environmental quality and preterm birth**.

Domain		Air		Water		Land		Built
Water	Water	0.003 (−0.002, 0.007)						
	Air	0.017 (0.013, 0.022)						
	IC	−0.005 (−0.010, 0.001)						
	Net effect	0.015 (0.011, 0.019)						
Land	Land	−0.007 (−0.011, −0.003)	Land	−0.001 (−0.005, 0.004)				
	Air	0.018 (0.013, 0.022)	Water	−0.013 (−0.017, −0.008)				
	IC	−0.003 (−0.009, 0.002)	IC	0.000 (−0.006, 0.006)				
	Net effect	0.008 (0.003, 0.012)	Net effect	−0.013 (−0.017, −0.010)				
Built	Built	**−0.005 (−0.009, −0.001)**	Built	0.001 (−0.003, 0.005)	Built	−0.011 (−0.015, −0.007)		
	Air	**0.021 (0.016, 0.026)**	Water	−0.009 (−0.013, −0.005)	Land	−0.011 (−0.015, −0.007)		
	IC	**−0.008 (−0.015, −0.002)**	IC	−0.004 (−0.010, 0.001)	IC	0.003 (−0.003, 0.009)		
	Net effect	**0.008 (0.004, 0.011)**	Net effect	−0.012 (−0.016, −0.009)	Net effect	−0.020 (−0.023, −0.016)		
SD	SD	**−0.012 (−0.017, −0.008)**	SD	−0.004 (−0.008, 0.000)	SD	**−0.016 (−0.021, −0.011)**	SD	−0.011 (−0.016, −0.007)
	Air	**0.022 (0.017, 0.027)**	Water	−0.025 (−0.029, −0.020)	Land	**−0.027 (−0.031, −0.022)**	Built	−0.021 (−0.025, −0.016)
	IC	**−0.013 (−0.020, −0.007)**	IC	0.003 (−0.002, 0.009)	IC	**0.013 (0.006, 0.019)**	IC	0.000 (−0.006, 0.007)
	Net effect	**−0.004 (−0.007, 0.000)**	Net effect	−0.025 (−0.029, −0.021)	Net effect	**−0.030 (−0.033, −0.026)**	Net effect	−0.032 (−0.036, −0.028)

*Models with interactions for pairwise EQI domain-specific indices for non-stratified analyses. Effect estimates shown are for worse environment (3rd tertile) compared to better (1st tertile). Estimates with significant interactions (IC *p*-value <0.05) are bolded*.

*^a^Effects shown are main effect for domain A, main effect for domain B, interaction term A × B, and net effect A + B + A × B. Models adjusted for percent minority and all other EQI domains*.

*IC, interaction contrast; SD, sociodemographic*.

**Table 3:** **Prevalence differences^a^ (95% confidence interval) for environmental quality and preterm birth**.

Domain		Air		Water		Land		Built
Water	Water	−0.001 (−0.007, 0.005)						
	Air	0.005 (−0.001, 0.012)						
	IC	−0.003 (−0.011, 0.006)						
	Net effect	0.002 (−0.004, 0.008)						
Land	Land	−0.008 (−0.015, −0.002)	Land	−0.003 (−0.010, 0.004)				
	Air	0.007 (0.001, 0.014)	Water	−0.012 (−0.018, −0.005)				
	IC	−0.003 (−0.012, 0.006)	IC	0.000 (−0.010, 0.009)				
	Net effect	−0.004 (−0.010, 0.002)	Net effect	−0.015 (−0.020, −0.010)				
Built	Built	−0.014 (−0.023, −0.006)	Built	0.000 (−0.006, 0.006)	Built	−0.011 (−0.017, −0.004)		
	Air	0.004 (−0.007, 0.014)	Water	−0.014 (−0.020, −0.008)	Land	−0.014 (−0.020, −0.008)		
	IC	0.002 (−0.011, 0.016)	IC	−0.003 (−0.012, 0.005)	IC	−0.004 (−0.012, 0.005)		
	Net effect	−0.008 (−0.013, −0.004)	Net effect	−0.017 (−0.024, −0.011)	Net effect	−0.028 (−0.034, −0.022)		
SD	SD	0.017 (0.011, 0.023)	SD	0.000 (−0.006, 0.006)	SD	**−0.006 (−0.013, 0.000)**	SD	−0.015 (−0.022, −0.009)
	Air	0.002 (−0.005, 0.008)	Water	0.021 (0.015, 0.027)	Land	**0.023 (0.017, 0.030)**	Built	0.023 (0.017, 0.029)
	IC	0.003 (−0.005, 0.011)	IC	−0.006 (−0.014, 0.003)	IC	**−0.016 (−0.024, −0.007)**	IC	−0.003 (−0.012, 0.005)
	Net effect	0.022 (0.015, 0.029)	Net effect	0.016 (0.009, 0.022)	Net effect	**0.002 (−0.006, 0.009)**	Net effect	0.004 (−0.002, 0.011)

*Models with interactions for pairwise EQI domain-specific indices for metropolitan urbanized counties. Effect estimates shown are for worse environment (3rd tertile) compared to better (1st tertile). Estimates with significant interactions (IC *p*-value <0.05) are bolded*.

*^a^Effects shown are main effect for domain A, main effect for domain B, interaction term A × B, and net effect A + B + A × B. Models adjusted for percent minority and all other EQI domains*.

*IC, interaction contrast; SD, sociodemographic*.

**Table 4:** **Prevalence differences^a^ (95% confidence interval) for environmental quality and preterm birth**.

Domain		Air		Water		Land		Built
Water	Water	**0.003 (−0.006, 0.011)**						
	Air	**0.021 (0.011, 0.030)**						
	IC	**−0.013 (−0.025, 0.000)**						
	Net effect	**0.011 (0.002, 0.019)**						
Land	Land	**0.006 (−0.003, 0.014)**	Land	−0.005 (−0.014, 0.004)				
	Air	**0.023 (0.014, 0.032)**	Water	−0.006 (−0.014, 0.003)				
	IC	**−0.012 (−0.025, 0.000)**	IC	−0.002 (−0.014, 0.011)				
	Net effect	**0.016 (0.008, 0.024)**	Net effect	−0.012 (−0.020, −0.004)				
Built	Built	−0.003 (−0.011, 0.006)	Built	−0.006 (−0.014, 0.003)	Built	−0.002 (−0.011, 0.008)		
	Air	0.019 (0.011, 0.028)	Water	−0.010 (−0.018, −0.001)	Land	0.003 (−0.005, 0.011)		
	IC	−0.007 (−0.020, 0.005)	IC	−0.003 (−0.015, 0.009)	IC	−0.010 (−0.022, 0.002)		
	Net effect	0.009 (−0.001, 0.019)	Net effect	−0.018 (−0.026, −0.010)	Net effect	−0.008 (−0.016, 0.000)		
SD	SD	**−0.004 (−0.013, 0.004)**	SD	−0.006 (−0.015, 0.002)	SD	−0.001 (−0.010, 0.009)	SD	−0.011 (−0.021, −0.001)
	Air	**0.024 (0.015, 0.033)**	Water	−0.021 (−0.030, −0.011)	Land	−0.013 (−0.023, −0.003)	Built	−0.018 (−0.027, −0.008)
	IC	**−0.017 (−0.029, −0.005)**	IC	0.007 (−0.006, 0.019)	IC	−0.004 (−0.017, 0.010)	IC	0.004 (−0.010, 0.017)
	Net effect	**0.003 (−0.006, 0.011)**	Net effect	−0.020 (−0.029, −0.012)	Net effect	−0.017 (−0.025, −0.010)	Net effect	−0.025 (−0.034, −0.017)

*Models with interactions for pairwise EQI domain-specific indices for non-metro urbanized counties. Effect estimates shown are for worse environment (3rd tertile) compared to better (1st tertile). Estimates with significant interactions (IC *p*-value <0.05) are bolded*.

*^a^Effects shown are main effect for domain A, main effect for domain B, interaction term A × B, and net effect A + B + A × B. Models adjusted for percent minority and all other EQI domains*.

*IC, interaction contrast; SD, sociodemographic*.

**Table 5:** **Prevalence differences^a^ (95% confidence interval) for environmental quality and preterm birth**.

Domain		Air		Water		Land		Built
Water	Water	−0.001 (−0.006, 0.005)						
	Air	0.016 (0.010, 0.022)						
	IC	−0.004 (−0.012, 0.005)						
	Net effect	0.012 (0.006, 0.018)						
Land	Land	−0.003 (−0.009, 0.004)	Land	−0.003 (−0.009, 0.003)				
	Air	0.020 (0.014, 0.026)	Water	−0.011 (−0.018, −0.005)				
	IC	−0.005 (−0.013, 0.003)	IC	0.003 (−0.006, 0.011)				
	Net effect	0.012 (0.005, 0.019)	Net effect	−0.012 (−0.018, −0.006)				
Built	Built	**0.005 (−0.001, 0.011)**	Built	−0.006 (−0.012, 0.001)	Built	−0.009 (−0.016, −0.002)		
	Air	**0.024 (0.018, 0.031)**	Water	−0.006 (−0.012, 0.001)	Land	−0.002 (−0.008, 0.005)		
	IC	**−0.015 (−0.024, −0.007)**	IC	0.001 (−0.008, 0.009)	IC	−0.001 (−0.010, 0.008)		
	Net effect	**0.014 (0.008, 0.021)**	Net effect	−0.010 (−0.016, −0.005)	Net effect	−0.012 (−0.018, −0.006)		
SD	SD	**−0.011 (−0.018, −0.004)**	SD	**−0.011 (−0.017, −0.006)**	SD	**−0.012 (−0.020, −0.004)**	SD	−0.004 (−0.011, 0.003)
	Air	**0.023 (0.017, 0.029)**	Water	**−0.027 (−0.034, −0.020)**	Land	**−0.035 (−0.045, −0.024)**	Built	−0.022 (−0.030, −0.015)
	IC	**−0.015 (−0.023, −0.006)**	IC	**0.012 (0.003, 0.020)**	IC	**0.019 (0.006, 0.033)**	IC	0.003 (−0.007, 0.013)
	Net effect	**−0.002 (−0.009, 0.004)**	Net effect	**−0.027 (−0.033, −0.020)**	Net effect	**−0.027 (−0.032, −0.022)**	Net effect	−0.023 (−0.029, −0.018)

*Models with interactions for pairwise EQI domain-specific indices for less urbanized counties. Effect estimates shown are for worse environment (3rd tertile) compared to better (1st tertile). Estimates with significant interactions (IC *p*-value <0.05) are bolded*.

*^a^Effects shown are main effect for domain A, main effect for domain B, interaction term A × B, and net effect A + B + A × B. Models adjusted for percent minority and all other EQI domains*.

*IC, interaction contrast; SD, sociodemographic*.

**Table 6:** **Prevalence differences^a^ (95% confidence interval) for environmental quality and preterm birth**.

Domain		Air		Water		Land		Built
Water	Water	0.002 (−0.011, 0.015)						
	Air	0.018 (0.005, 0.031)						
	IC	−0.007 (−0.025, 0.010)						
	Net effect	0.013 (0.001, 0.024)						
Land	Land	**0.009 (−0.005, 0.022)**	Land	−0.005 (−0.018, 0.008)				
	Air	**0.035 (0.022, 0.048)**	Water	−0.016 (−0.029, −0.002)				
	IC	**−0.028 (−0.048, −0.008)**	IC	0.009 (−0.009, 0.026)				
	Net effect	**0.015 (−0.002, 0.033)**	Net effect	−0.012 (−0.026, 0.003)				
Built	Built	**0.012 (0.000, 0.024)**	Built	−0.005 (−0.018, 0.008)	Built	−0.009 (−0.022, 0.005)		
	Air	**0.029 (0.018, 0.041)**	Water	−0.003 (−0.016, 0.009)	Land	−0.010 (−0.022, 0.003)		
	IC	**−0.024 (−0.041, −0.006)**	IC	0.004 (−0.014, 0.021)	IC	0.007 (−0.010, 0.025)		
	Net effect	**0.018 (0.004, 0.031)**	Net effect	−0.004 (−0.015, 0.007)	Net effect	−0.011 (−0.023, 0.001)		
SD	SD	**−0.003 (−0.017, 0.011)**	SD	0.003 (−0.010, 0.016)	SD	−0.009 (−0.024, 0.005)	SD	0.002 (−0.013, 0.017)
	Air	**0.030 (0.017, 0.043)**	Water	−0.015 (−0.028, −0.001)	Land	−0.016 (−0.032, 0.000)	Built	−0.002 (−0.016, 0.012)
	IC	**−0.027 (−0.045, −0.009)**	IC	−0.007 (−0.024, 0.011)	IC	0.003 (−0.019, 0.025)	IC	−0.017 (−0.037, 0.002)
	Net effect	**0.000 (−0.015, 0.016)**	Net effect	−0.018 (−0.032, −0.004)	Net effect	−0.023 (−0.033, −0.012)	Net effect	−0.018 (−0.029, −0.006)

*Models with interactions for pairwise EQI domain-specific indices for thinly populated counties. Effect estimates shown are for worse environment (3rd tertile) compared to better (1st tertile). Estimates with significant interactions (IC *p*-value <0.05) are bolded*.

*^a^Effects shown are: main effect for domain A, main effect for domain B, interaction term A × B, net effect A + B + A × B. Models adjusted for percent minority and all other EQI domains*.

*IC, interaction contrast; SD, sociodemographic*.

The majority of main effects for the non-urban/rural-stratified models were positive (indicating an increase in PTB prevalence with decreasing environmental quality) for the air domain index and negative for the other domain indices (Table 2). Antagonistic interactions were seen between the sociodemographic/air domains [net effect PD: −0.004 (95% CI: −0.007, 0.000), interaction contrast PD: −0.013 (95% CI: −0.020, −0.007)] and built/air domains [net effect PD: 0.008 (95% CI 0.004, 0.011), interaction contrast PD: −0.008 (95% CI: −0.015, −0.002)]. Antagonistic interaction was seen between the sociodemographic and land domains [net effect PD: −0.030 (95% CI: −0.033, −0.026), interaction contrast PD: 0.013 (95% CI: 0.020, 0.007)]. Note that since sociodemographic and land main effects were negative (sociodemographic PD: −0.016 and land PD: −0.027) and the interaction contrast was positive, the interaction is antagonistic, i.e., interaction is closer to the null than you would expect when adding the two main effects (expected strictly additive interaction: −0.016 + −0.027 = −0.043). All other interactions were non-significant, indicating a strictly additive relationship between the two modeled domains.

Evidence of antagonistic interaction was found across all rural/urban stratified analyses. Antagonistic interaction was found in metropolitan urbanized counties between sociodemographic and land domains [net effect PD: 0.002 (95% CI: −0.006, 0.009), interaction contrast PD: −0.016 (95% CI: −0.024, −0.007)] (Table 3). Antagonistic interaction was found in non-metro urbanized counties between the air domain with water, land, and sociodemographic domains (Table 4). In the less urbanized counties antagonistic relationships were found between air/built domains [net effect PD: 0.014 (95% CI: 0.008, 0.021), interaction contrast PD: −0.015 (95% CI: −0.024, −0.007)]; air/sociodemographic domains [net effect PD: −0.002 (95% CI: −0.009, 0.004), interaction contrast PD: −0.015 (95% CI: −0.023, −0.006)], as well as the sociodemographic/water domains and air domains (Table 5). In the thinly populated counties, antagonism was between the air domain and the land, and built and sociodemographic domains (Table 6).

Table 7 displays the summary of the interactions found across all domains and urban/rural-stratified models. Across all of the 50 total models, 15 models suggested interactions which were a departure from strict additivity. These interaction contrasts from Tables 2–6 all indicated antagonistic interaction.

**Table 7:** **Summary of interaction between domain interactions, worse (3rd tertile) compared to better (1st tertile)**.

		Non-stratified	Metropolitan urbanized	Non-metro urbanized	Less urbanized	Thinly populated
Domain A	Domain B					
Water	Air			Antagonism		
Land						
	Air			Antagonism		Antagonism
	Water					
Built	Air	Antagonism			Antagonism	Antagonism
	Water					
	Land					
SD	Air	Antagonism		Antagonism	Antagonism	Antagonism
	Water				Antagonism	
	Land	Antagonism	Antagonism		Antagonism	
	Built					

*SD, sociodemographic*.

## Discussion

We used PTB as a motivating example for the analysis of interaction effects. The main effects observed herein were mostly positive across the air domain and negative across the other domains. This is similar to previously published work, in which worsening air quality was associated with increased PTB, while worsening quality in other environmental domains was not associated with PTB or was associated with decreased PTB; in that work, associations did vary by urban/rural strata, with worsening sociodemographic quality associated with increased PTB only in the metropolitan urbanized counties (13).

The majority of models did not have significant interactions, indicating primarily additive relationships between any two domains. We found some evidence for antagonistic relationships between environmental domains, many of which yielded net effects nearer to the null than one would expect from strictly additive effect. Variations in the number of interactions and interacting domains were observed across urban/rural strata. More interactions were found in the less dense counties (less urbanized and thinly populated) as compared to more urban strata.

Across levels of urbanicity, the number and combinations of domain interactions differed across stratum, although all were antagonistic. In metropolitan urbanized counties interaction was observed between the sociodemographic/land domains only. We may see fewer interactions in highly urban regions because of potentially strong heterogeneity between areas of good and bad environments. In non-metro urbanized counties, interaction was observed in the air domain with water, land, and sociodemographic domains. In the less urbanized counties, interactions were found between air/built domains, air/sociodemographic, sociodemographic/water domains, and sociodemographic/land domains. In the thinly populated counties, interaction was found between the air domain with the land, built, and sociodemographic domains.

Antagonistic relationships were observed more often in the less dense counties (less urbanized and thinly populated); it is possible that with respect to their underlying populations and environments, extremely urban areas are more similar to one another, while most rural areas may be more heterogeneous. Interactions in the sociodemographic and built domains were seen across urban/rural status. These differences in urbanicity may be partially explained by the variables included in the sociodemographic domain. These variables (e.g., percent of housing with more than 10 units, percent earning greater than high school education, etc.) may better characterize urban poverty/sociodemographic status because factors such as education may be less meaningful in terms of economic success in a primarily farming community. In the three less urban strata, the air domain frequently has antagonistic associations with the other domains. This could be due in part to the strength of the associations of the air domain with PTB and that the air domain had positive associations with PTB, while the other domains were negatively associated.

In addition, differences in urban/rural strata may be explained through differing contributions of variable loadings. In PCA, variable loadings represent the strength of a variable’s contribution to the index value, which vary by urbanicity in the EQI. For example, in the sociodemographic domain index, one factor included in the index, the percent of people at or below poverty level, loaded highly positive in the metropolitan urban stratum and highly negative for all other strata (2). The previously published studies on EQI and PTB discuss the variation in variable loadings across urban/rural status, particularly in the sociodemographic domain (13). Urban environments were more likely to have complete spatial and temporal data within the original variables of the EQI (2). Estimates in the urban strata may be less likely to be biased compared to rural strata. These details might also help explain why we saw null total effects even when domain main effects indicated relationships with PTB.

Observed antagonistic interactions could have several possible explanations. PCA ranks each county based on variables used to represent each domain; since we do expect that counties will rank similarly across domains, looking at interactions between indices may be less informative than combining quantitative metrics (e.g., individual pollutants) of environmental exposure into a single metric, as in the overall EQI. As each index includes both beneficial and detrimental aspects of the environment, county rankings might be slightly unbalanced due to the even weighting of all factors when including multiple indices. For instance, an index which includes 10 beneficial and 10 detrimental aspects of environment will be a more balanced total picture of quality, then an index with only detrimental aspects especially when compared side by side. They both create an environmental metric, but one would estimate total environment and one would better estimate bad environment creating potential unexpected interaction relationships. The eigenvalues from the PCA used to construct the domains were equally scaled and centered, which should balance some of these differences. If in truth some factors are more influential in health then others, then this magnitude of effect is lost, especially in comparing domains to each other. The overall EQI, which uses a secondary PCA decomposition, might better account for the variation in measurement across domains.

While this study explores a complex area of environmental interaction, there are potential limitations. PTB is a useful indicator of national health; however, modes of action/mechanisms from environmental quality leading to PTB have not been well established, though some possibilities have been put forth (28). A clearer understanding of the pathways acting from environmental quality to PTB could be utilized to identify potential biologic interactions along with observed statistical interactions. The nature of the EQI and the underlying data leads to potential smoothing of exposure. In other words, some exposures may change over a smaller spatial or temporal scale than the county-level and 5-year period used, which would be homogenized by using the EQI. However, the cumulative nature of the EQI does account for simultaneous exposures.

To our knowledge, no other study has examined interaction between different environmental domains, and we were additionally able to examine this at the county-level across the entire United States. As the EQI is a publically available resource in the assessment of environmental quality, other studies may consider using the EQI to control for environmental factors (15) or to assess interaction between exposures and environmental quality. We assessed additive interaction. While we did observe some departures from additivity, many observed effects were strictly additive. In terms of public health importance, this strict additive interaction indicates that those living in areas influenced by multiple poor environmental factors may be at higher risk of PTB than those in an area where a single environmental domain is of poor quality, as one might expect. However, the antagonistic relationships indicate that this may not always hold true in rural areas.

When evaluating environmental exposures on health outcomes, vigilance in testing both traditional confounding and interaction should be exercised as we know exposures occur naturally simultaneously. This study was the first to explore interactions across different environmental domains and demonstrates the utility of the EQI to examine the relationship between environmental domain interactions and human health. Our results demonstrated the existence of interaction between EQI domains, and this application should be considered in future environmental analyses.

## Author Contributions

SG contributed to the study conception, performed statistical analyses, and drafted the manuscript. KR contributed to the construction of the EQI, performed statistical analyses, and helped to draft and edit the manuscript. CG contributed to the interpretation of the data and provided critical revisions to the manuscript. JJ contributed to the construction of the EQI and provided critical revisions to the manuscript revisions. YJ contributed to statistical analyses and provided critical revisions to the manuscript. LM constructed the EQI and provided critical revisions to the manuscript. DL conceived of the EQI, oversaw its design and coordination, oversaw coordination of this study, and contributed to manuscript revisions. All authors read and approved the final manuscript and agree to be accountable for all aspects of this work.

## Disclaimer

The research described in this article has been reviewed by the National Health and Environmental Effects Research Laboratory, U.S. Environmental Protection Agency, and approved for publication. Approval does not signify that the contents necessarily reflect the views and policies of the Agency nor does the mention of trade names of commercial products constitute endorsement or recommendation for use.

## Conflict of Interest Statement

The authors declare that the research was conducted in the absence of any commercial or financial relationships that could be construed as a potential conflict of interest.

## Abbreviations

CI, confidence interval; EQI, environmental quality index; PCA, principal component analysis; PDs, prevalence differences; PTB, preterm birth.
